# Variations in Autophagy-Related Genes *ATG5, ATG10* and *ATG16L1* Correlate with Tumor Burden, Inflammatory Biomarkers and Clinical Course in Patients with Metastatic Melanoma Treated with Immune Checkpoint Inhibitors

**DOI:** 10.3390/cancers18162709

**Published:** 2026-08-21

**Authors:** Milica Ćućuz Jokić, Bojana Cikota-Aleksić, Jovana Pavlica, Branko Dujović, Igor Salatić, Tijana Stanojković, Tatjana Bollhorn, Lidija Kandolf

**Affiliations:** 1 Institute of Medical Research, Military Medical Academy, 11000 Belgrade, Serbia; 2 Centre for Clinical Pharmacology, Military Medical Academy, 11000 Belgrade, Serbia; stanojkovictijana96@hotmail.com; 3 Clinic for Dermatovenerology, Military Medical Academy, 11000 Belgrade, Serbia; pavlicajovana23@gmail.com (J.P.); brankodujovic@yahoo.com (B.D.); igor.salatic@gmail.com (I.S.); lkandolfsekulovic@gmail.com (L.K.); 4 Faculty of Medicine of the Military Medical Academy, University of Defense, 11000 Belgrade, Serbia; 5 Institute of Forensic Medicine, Faculty of Medicine, University of Belgrade, 11000 Belgrade, Serbia; tatjana.varljen@med.bg.ac.rs

**Keywords:** melanoma, autophagy, autophagy related genes, single nucleotide polymorphisms, immunotherapy

## Abstract

**Background/Objectives**: This study assessed the impact of variations in autophagy-related genes (*ATG*) on baseline characteristics of cutaneous melanoma, laboratory parameters (including inflammatory biomarkers), response to therapy, and survival in patients treated with immune checkpoint inhibitors (ICIs) as first-line therapy. **Methods**: DNA was extracted from blood samples of 144 melanoma patients. Genotyping of *ATG5* (rs2245214 and rs510432), *ATG10* (rs1864183 and rs1864182), and *ATG16L1* (rs2241880) was performed using an allelic discrimination method on the StepOnePlus^TM^ Real-Time PCR System. Correlations with laboratory parameters, response to therapy, and survival were assessed only in the subgroup of patients who received ICIs in first-line treatment (*n* = 74). Statistical significance was calculated, and *p* values were adjusted for multiple testing using the Benjamini–Hochberg False Discovery Rate (FDR). **Results**: Considering baseline characteristics of 144 patients, *ATG5* rs2245214 showed a trend with regression (*p* = 0.042) and lymphovascular invasion (*p* = 0.05), while *ATG16L1* rs2241880 was associated with lymphovascular invasion (*p* = 0.056), with corrected FDR q value for all histopathological characteristics of 0.076. In patients who received ICIs in first-line, *ATG5* rs2245214 genotypes were associated with LDH (*p* = 0.001, q = 0.004) and the number of metastatic sites (*p* < 0.001, q = 0.004). Also, *ATG5* rs2245214 was associated with neutrophil-to-lymphocyte ratio (NLR) (*p* = 0.036, q = 0.045) systemic immune-inflammation (SII) index (*p* = 0.038, q = 0.048) and pan-immune-inflammation value (PIV) (*p* = 0.031,q = 0.041), while *ATG10* rs1864183 was associated with PIV (*p* = 0.047, q = 0.047). The association of *ATG* genotypes with disease control rate (DCR) was demonstrated for *ATG5* rs2245214 (*p* = 0.013, q = 0.029) and *ATG16L1* rs2241880 (*p* = 0.032,q = 0.032). Progression-free survival (PFS) was significantly associated with *ATG10* rs1864183 (*p* = 0.023, q = 0.046). The significance of the *ATG10* rs1864183 C/T genotype as a prognostic marker for progression was confirmed in both univariate and multivariate Cox proportional hazards regression analyses (*p* = 0.025, q = 0.028 and *p* = 0.022, respectively). **Conclusions**: This study shows that *ATG5* rs2245214, *ATG10* rs1864183, and *ATG16L1* rs2241880 correlate with systemic inflammation, response to ICIs, and had a trend toward melanoma characteristics. However, these findings should be confirmed in larger patient cohorts.

## Simple Summary

Autophagy is an evolutionarily conserved process by which cells degrade damaged organelles, misfolded proteins, and pathogens to maintain homeostasis. Its role in cancer may be contradictory or context-dependent. However, the findings that autophagy supports tumor progression and contributes to therapy resistance underscore the importance of identifying autophagy-related markers with predictive/prognostic potential. This study analyzed the association between variations in the autophagy-related genes *ATG5*, *ATG10*, and *ATG16L1* and the characteristics/clinical course of cutaneous melanoma. Our findings suggest a trend between *ATG* genotypes and the clinical/pathologic characteristics of melanoma and indicate an association between *ATG* genotypes and systemic inflammation indexes, response to immunotherapy when used as first-line treatment, and time to progression; however, these results require validation in larger patient cohorts.

## 1. Introduction

Melanoma is an aggressive tumor of melanocytes that predominantly develops on the skin. Given the continuously rising incidence and mortality rates worldwide, it represents a significant health and economic burden on society [1]. In advanced stages (unresectable or metastatic disease), melanoma is associated with a poor prognosis. However, outcomes have been improved significantly with the introduction of targeted therapy (BRAF and MEK inhibitors). Additionally, immune checkpoint inhibitors (ICIs) have further advanced the treatment of patients with melanoma [2]. ICIs mainly include drugs that block the programmed death-1 (PD-1) receptor (e.g., nivolumab, pembrolizumab), inhibit programmed death-ligand-1 (PD-L1) (e.g., atezolizumab), or inhibit cytotoxic T-lymphocyte antigen-4 (CTLA-4) (e.g., ipilimumab) [3].

Despite improved outcomes, a proportion of patients display resistance to therapy, experience adverse effects, or relapse after an initially successful response. Therefore, reliable markers to identify patients who will (or will not) benefit from ICIs or develop side effects are needed in the clinical setting.

Previous studies have indicated that elevated serum lactate dehydrogenase (LDH) level, more than three metastatic sites, and an Eastern Cooperative Oncology Group (ECOG) performance status of ≥1 are markers of poor outcome in advanced melanoma treated with targeted therapy. To define predictive biomarkers for ICIs, the characteristics of the tumor, tumor microenvironment, and host have been extensively studied. Serum LDH and PD-L1 expression have been suggested as potential markers for therapy selection (e.g., monotherapy vs. combination of ICIs). However, no predictive biomarkers for the efficacy of ICIs have been adopted in routine clinical practice to date [4].

Several studies have identified autophagy as a key mechanism of resistance to BRAF or BRAF-MEK inhibitors in BRAF V600-positive tumors. Autophagy is a highly regulated cellular process in which cytoplasmic components are sequestered and degraded by lysosomes [5]. This process supports cellular homeostasis and quality control and can be triggered by environmental or intracellular signals, including tissue damage caused by therapy or radiation. The role of autophagy in tumors appears to be context-dependent; it may be tumor-promoting or tumor-suppressive, depending on cooperating genetic alterations, tumor stage/grade, and the microenvironment [6,7]. In addition, autophagy participates in the differentiation and function of lymphoid and myeloid cells, affects innate and adaptive immunity, and can reshape the tumor microenvironment [8]. Therefore, the impact of autophagy on the response to ICIs is of particular interest, and some previous findings have implicated suppression of autophagy as a contributor to resistance to CTLA-4 blockade in melanoma [9]. There are attempts to modulate autophagic flux in the tumor microenvironment by different approaches (small molecules, different drugs, nanoparticles) and improve immunotherapy outcomes [10]. In the preclinical models of non-small cell lung cancer, inhibition of autophagy enhances the efficacy of anti-PD1 therapy [11].

The process of autophagy is precisely regulated by the coordinated action of at least 69 autophagy-related (ATG) genes and proteins [12]. Previous reports indicated the association of the *ATG5*, *ATG10* and *ATG16L1* single-nucleotide polymorphisms (SNPs) with histopathological and clinical characteristics (e.g., clinical stage, melanoma localization; Breslow thickness, age at diagnosis) [13]. Therefore, the present study also assessed the association between five SNPs in *ATG5*, *ATG10* and *ATG16L1* and relevant laboratory, histopathological, and clinical characteristics of melanoma patients. Furthermore, we analyzed the impact of *ATG5*, *ATG10* and *ATG16L1* SNPs on response to ICIs and clinical outcome in patients with advanced/metastatic melanoma.

## 2. Materials and Methods

### 2.1. Patients

The present study included 144 patients with cutaneous melanoma who were treated and followed up at the Clinic for Dermatovenerology, Military Medical Academy, Belgrade, Serbia. Exclusion criteria for participation in the study were age under 18 years, presence of systemic inflammatory diseases and/or other malignancies, and insufficient follow-up (<24 months).

Survival analysis included only patients with unresectable stage III/IV disease treated with ICIs as first-line therapy (pembrolizumab, nivolumab, or ipilimumab plus nivolumab regimen) (*n* = 74). Response to therapy was evaluated according to the Response Evaluation Criteria in Solid Tumors (RECIST v1.1) [14].

The study design was approved by the Ethics Committee of the Military Medical Academy (Approval No 43/2022). All patients signed a consent form to participate in the study after they were informed about the planned research.

### 2.2. DNA Extraction and Genotyping

Blood samples were collected in EDTA tubes at the baseline time point (before the start of the systemic therapy) and kept at −20 °C. DNA was extracted using the MagCore^®^ Genomic DNA Whole Blood Kit, Cartridge Code 102 (RBC Bioscience, RBC Bioscience, New Taipei City, Taiwan) for the MagCore^®^ Plus II Automated Nucleic Acid Extractor (RBC Bioscience, China).

DNA samples were subjected to *ATG5* (rs2245214 and rs510432), *ATG10* (rs1864183, rs1864182), and *ATG16L1* (rs2241880) genotyping by an allelic discrimination method on the StepOnePlus^TM^ Real Time PCR System (Applied Biosystems, Thermo Fisher Scientific, Waltham, MA, USA). Genotyping was performed by usage of TaqMan™ SNP Genotyping Assays (assays IDs: C___3001905_20 for rs2245214; C____910351_10 for rs510432; C__11953871_20 for rs1864183; C__11953870_70 for rs1864182; C___9095577_20 for rs2241880) and TaqMan™ Genotyping Master Mix (both Thermo Fisher Scientific, Waltham, MA, USA).

### 2.3. Statistics

The baseline demographic and clinicopathological variables were summarized using descriptive statistics. The association between genotypes and clinical characteristics, response to therapy, relapse/progression, and therapy-related adverse events was evaluated across genetic models (dominant, recessive, codominant, and overdominant). Odds ratio (OR) and 95% confidence interval (CI) were calculated to estimate the relative risk regarding different genotypes.

Calculation of inflammatory biomarkers included absolute cell counts at baseline (within 30 days before therapy initiation) and lymphocyte-to-monocyte ratio (LMR), neutrophil-to-lymphocyte ratio (NLR), platelet-to-lymphocyte ratio (PLR), monocyte-to-lymphocyte ratio (MLR), pan-immune-inflammation value (PIV) (neutrophil count × platelet count × monocyte count)/lymphocyte count, and systemic immune-inflammation index (SII) (neutrophil count × platelet count)/lymphocyte count. All baseline absolute cell counts were in 10^9^/L.

The cut-off values were calculated using ROC curves to predict survival of “>1 year”, selecting the value that maximizes the distance from the diagonal [15].

Progression-free survival (PFS) was defined as the time from the start of treatment to radiologically confirmed disease progression per RECIST v1.1 criteria or to death from any cause. When a new therapy was started before progression or death, patients were censored at the start of the next therapy. Patients without progression or death and no further therapies were censored at the date of last contact. Overall survival (OS) was calculated as the time from the start of treatment to death from any cause or to the last contact. Patients without a recorded date of death were censored at the date of their last known contact. Survival analyses included only patients treated with systemic immunotherapy. PFS and OS curves were presented using the Kaplan–Meier method.

*p* values were calculated using Fisher’s exact test or Chi-Squared test for categorical variables, Student’s *t*-test for continuous variables, and the log-rank test for Kaplan–Meier estimates.

The most prominent factors for progression and survival were identified using logistic regression and Cox proportional hazards regression.

Benjamini–Hochberg False Discovery Rate procedure was used to correct for multiple testing, and q < 0.05 was considered statistically significant. FDR correction was performed separately for clinical/pathological characteristics (6 tests), inflammatory/laboratory parameters (15 tests), response to ICI therapy (5 tests), survival outcomes (2 tests), and univariate Cox proportional hazards screening (10 tests). All analyses were performed by snpStats (1.22.0.) [16] and EZR 1.36 (Saitama Medical Center Jichi Medical University, Saitama, Japan) [17].

## 3. Results

### 3.1. Clinical Characteristics and Therapy

This study included 144 patients with cutaneous melanoma [53 females (36.81%) and 91 males (63.19%)], aged 19–87 years, with a median age of 61 years. The majority of patients presented with melanoma in clinical stage III (50%), with the most common stage being IIIC (29.86%). Melanoma in clinical stage IV was initially diagnosed in 14.58% of patients. A total of 74 patients [27 females (36.49%) and 47 males (63.51%)], aged 20–85 years (median 63.5 years), with unresectable melanoma in clinical stage III/IV, were treated with first-line immunotherapy. Histopathologic characteristics of the study population (*n* = 144) and of the subgroup of patients treated with ICIs as first-line therapy are summarized in Table 1.

As first-line immunotherapy, 36/74 (48.65%) of patients received pembrolizumab, 31/74 (41.89%) nivolumab, and 7/74 (9.46%) combination nivolumab/ipilimumab. At the start of immunotherapy, 17.57% of patients had more than three metastatic sites. Baseline LDH was elevated in 12 patients (16.22%), and S-100 was elevated in 27 (36.49%). The majority of patients (90.54%) had an ECOG performance status of 0 or 1.

### 3.2. Frequencies of Alleles and Genotypes

The observed allele and genotype frequencies for *ATG5* (rs2245214 and rs510432), *ATG10* (rs1864183 and rs1864182), and *ATG16L1* (rs2241880) are summarized in Supplementary Table S1. For each locus, genotype frequencies were in Hardy–Weinberg equilibrium (*p* > 0.05; Supplementary Table S1). Linkage disequilibrium was observed between *ATG5* rs2245214 and rs510432 (D′ = 0.71, r^2^ = 0.31, *p* < 0.001) and between *ATG10* rs1864183 and rs1864182 (D′ = 0.96, r^2^ = 0.71, *p* < 0.001).

### 3.3. Association of Alleles/Genotypes with Demographic, Clinical, and Histopathologic Characteristics

The association analyses included baseline demographic characteristics [age (≤60 vs. >60 years) and gender (male vs. female)], clinical characteristics [melanoma localization (head&neck vs. trunk vs. lower extremities vs. upper extremities)] and histopathologic characteristics [histologic type (superficial spreading melanoma vs. lentigo maligna melanoma vs. nodular melanoma vs. acral lentiginous melanoma vs. other), Breslow thickness (<4 mm vs. ≥4 mm), microsatellites (present vs. absent), ulcerations (present vs. absent), mitosis (≤5 vs. >5), tumor infiltrating lymphocytes (TIL) (present vs. absent), tumor regression (present vs. absent), lymphovascular invasion (present vs. absent), and perineural invasion (present vs. absent)].

Analyses using different genetic models demonstrated a borderline association of *ATG5* rs2245214 with regression (recessive model, *p* = 0.042) and lymphovascular invasion (dominant model, *p* = 0.05), both yielding an FDR q value of 0.076. Tumor regression was less frequent in patients with at least one *ATG5* rs2245214 G allele (C/G or G/G genotypes) than in C/C carriers (20.41% vs. 75%; OR 0.09, 95% CI 0.01–0.91), while lymphovascular invasion was less frequent in patients with C/G or G/G genotypes than in C/C carriers (7.31% vs. 25%; OR 0.24, 95% CI 0.06–0.98). Under the overdominant model, *ATG5* rs510432 demonstrated a trend toward an association with ulcerations (*p* = 0.06, q = 0.076). Patients with the C/T genotype were more likely to have a tumor with ulcerations than those with the C/C or T/T genotype (67.1% vs. 49.09%; OR 2.13, 95% CI 1–4.51). Similar borderline trends were observed for associations of *ATG16L1* rs2241880 with TIL (codominant model, *p* = 0.076, q = 0.076) and lymphovascular invasion (overdominant model, *p* = 0.056, q = 0.076), and of *ATG5* rs510432 with microsatellites (dominant model, *p* = 0.076, q = 0.076).

No other notable trends or associations with genotypes and baseline characteristics were found.

### 3.4. Clinical Impact of ATG Genotypes in Patients with Unresectable Stage III/IV Melanoma Receiving First-Line Immunotherapy

The analysis of associations between ATG genotypes and laboratory parameters (LDH, S-100), inflammatory biomarkers, number of metastatic sites, response to therapy, and survival (PFS and OS) included only patients with unresectable stage III/IV melanoma receiving ICIs as first-line therapy (*n* = 74). Values for LDH, S-100, inflammatory biomarkers, and the number of metastatic sites were obtained within 30 days prior to initiation of the first treatment cycle. The impact of *ATG* genotypes was analyzed using different genetic models (dominant, recessive, codominant, and overdominant).

#### 3.4.1. LDH, S-100, and the Number of Metastatic Sites

Analysis using codominant and overdominant genetic models showed significant associations between ATG5 rs2245214 genotypes and serum LDH levels and the number of metastatic sites. The frequency of the C/G genotype was significantly lower in patients with elevated serum LDH (*p* = 0.001 in both codominant and overdominant models), and in those with more than three metastatic sites (*p* < 0.001 in a codominant model, *p* = 0.001 in an overdominant model). After applying multiple testing corrections, the adjusted *p* levels (q levels) were 0.004 for the associations of ATG5 rs2245214 with LDH levels and with more than three metastatic sites. There was no significant association between *ATG* genotypes and S-100 levels.

#### 3.4.2. Inflammatory Biomarkers

**Figure 1:**
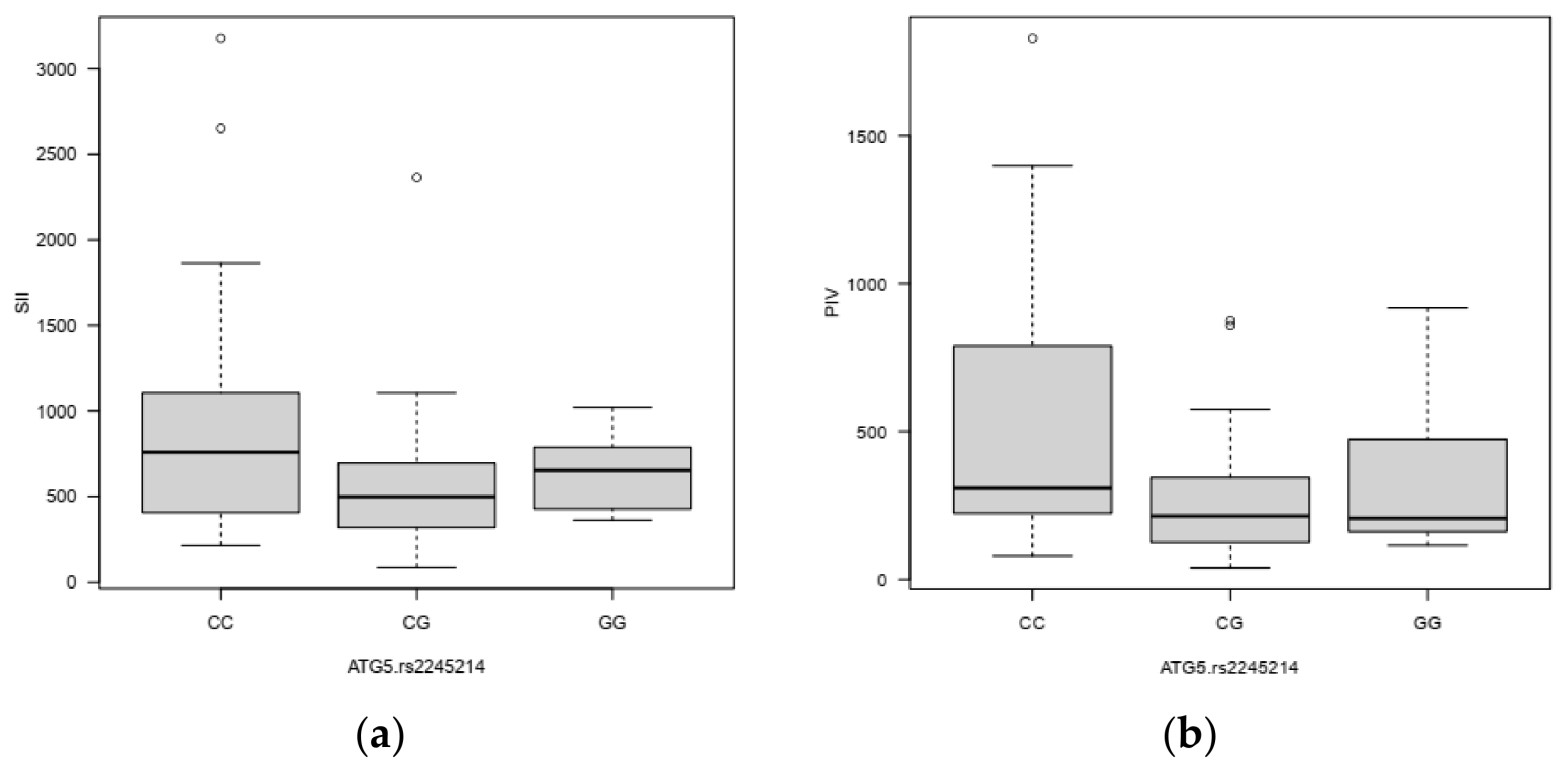

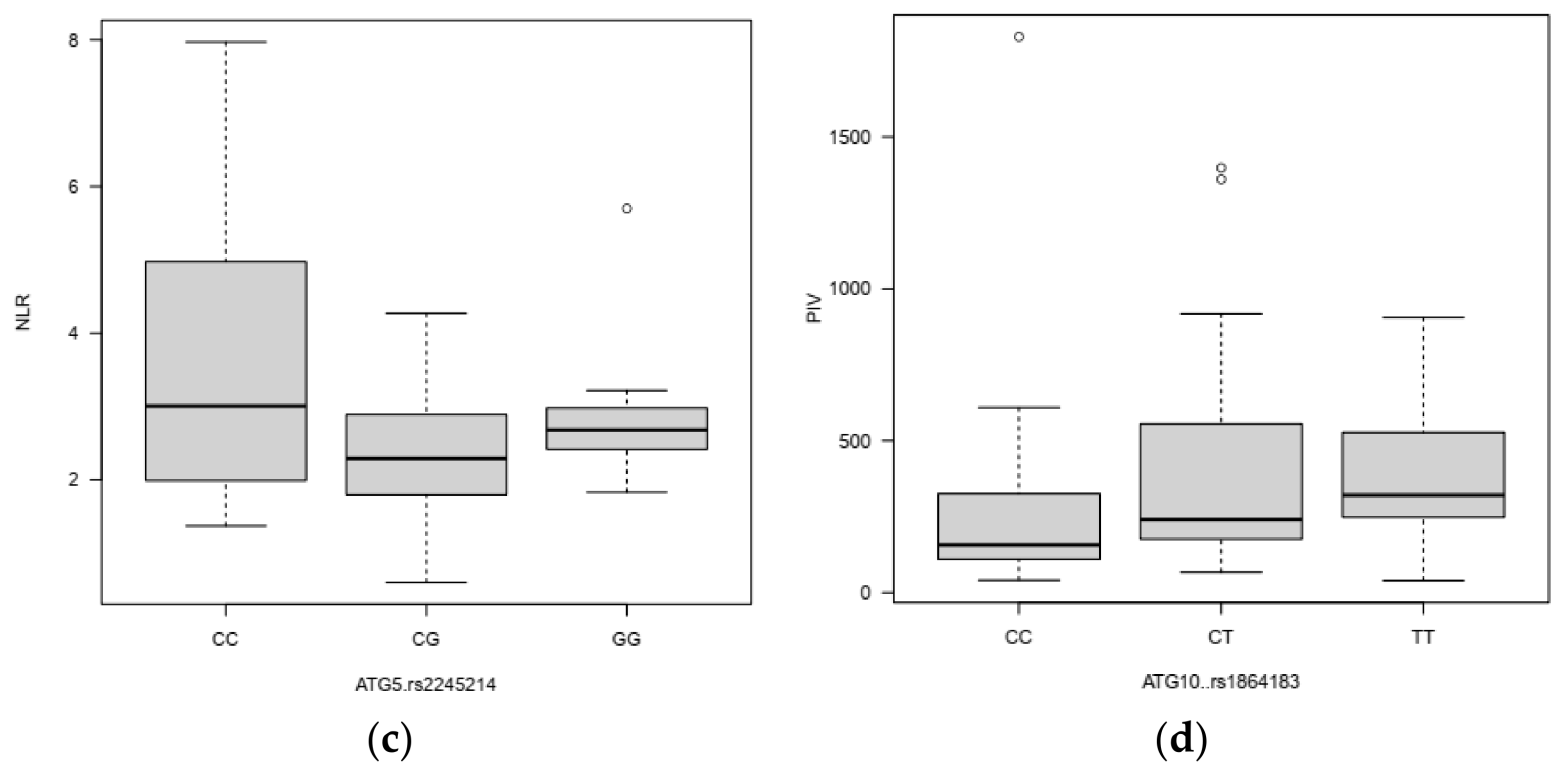
Inflammatory biomarkers in patients with different *ATG5* rs2245214 and *ATG10* rs1864183 geno-types. (**a**) SII indexes in patients with *ATG5* rs2245214 C/C (759.22), C/G (497.99), and G/G (654.28) genotypes; SII indexes differed significantly by *ATG5* rs2245214 genotype (codominant model, *p* = 0.038, q = 0.048). (**b**) PIV indexes in patients with *ATG5* rs2245214 C/C (309.75), C/G (214.44), and G/G (207.20) genotypes; PIV indexes differed significantly by *ATG5* rs2245214 genotype (codominant model, *p* = 0.031, q = 0.041). (**c**) NLR indexes in patients with *ATG5* rs2245214 C/C (3.01), C/G (2.29), and G/G (2.68) genotypes; NLR indexes differed significantly by *ATG5* rs2245214 genotype (co-dominant model, *p* = 0.036, q = 0.045). (**d**) PIV indexes in patients with *ATG10* rs1864183 C/C (157.76), C/T (240.97), and T/T (320.82) genotypes; PIV indexes differed significantly by *ATG10* rs1864183 genotype (codominant model, *p* = 0.047, q = 0.047).

An association between genotypes and systemic inflammation was observed for the *ATG5* rs2245214 and *ATG10* rs1864183. *ATG5* rs2245214 was associated with NLR (*p* = 0.036, q = 0.045 in a codominant model, *p* = 0.027, q = 0.039 in a dominant model, and *p* = 0.011, q = 0.029 in an overdominant model), SII (*p* = 0.038, q = 0.048 in a codominant model, *p* = 0.014, q = 0.030 in a dominant model, and *p* = 0.016, q = 0.030 in an overdominant model), and PIV (*p* = 0.031, q = 0.041 in a codominant model, *p* = 0.014, q = 0.030 in a dominant model, and *p* = 0.016, q = 0.030 in an overdominant model). Patients with the *ATG5* rs2245214 C/G genotype had the lowest NLR and SII, whereas PIV was similar between C/G and G/G carriers and significantly lower than in patients with the C/C genotype (Figure 1).

*ATG10* rs1864183 was associated with PIV (*p* = 0.047, q = 0.047 in a codominant model, *p* = 0.019, q = 0.032 in a dominant model), and patients with the C/C genotype had the lowest PIV index (Figure 1).

No statistically significant association was observed between other *ATG* genotypes and systemic inflammation indices (MLR, LMR, PLR, SII, and PIV).

#### 3.4.3. Response to Therapy

Data on response to ICIs as first-line therapy were available for 67/74 patients (90.54%). Response to therapy, presented as the disease control rate (DCR) or the objective response rate (ORR), is shown in Table 2.

A significant association of *ATG* genotypes with DCR was demonstrated for *ATG5* rs2245214 (*p* = 0.013, q = 0.029)in a codominant model, *p* = 0.007, q = 0.029 in a dominant model, and *p* = 0.017, q = 0.029 in an overdominant model) and *ATG16L1* rs2241880 (*p* = 0.032,q = 0.032 in a codominant model and *p* = 0.026,q = 0.032 in a dominant model) (Table 3). Among *ATG5* rs2245214 genotypes, the C/G genotype was associated with the highest rates of favorable DCR. For *ATG16L1* rs2241880, the most significant difference in DCR was observed in the G/G genotype.

Analysis through different genetic models did not reveal a significant correlation between *ATG* genotypes and ORR.

#### 3.4.4. Survival Analyses

**Figure 2:**
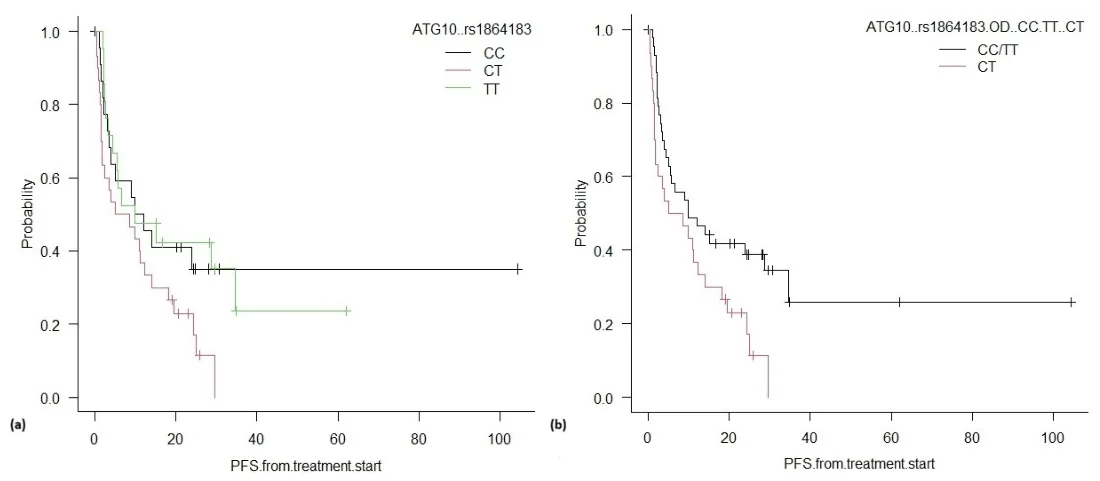
Progression-free survival (PFS) in patients treated with ICIs as first-line systemic immunotherapy regarding *ATG*10 *rs1864183* genotypes. (**a**) Codominant genetic model; median survival was 11.01 months (95% CI 3.22-NA), 6.8 months (95% CI 1.77–14.17), and 10.03 months (95% CI 3.4–34.57) for patients with *ATG*10 *rs1864183* C/C, C/T, and T/T genotype, respectively (*p* = 0.073, q = 0.073). (**b**) Overdominant genetic model; median survival was 10.03 months (95% CI 4.5–28.7) and 6.8 months (95% CI 1.77–14.17) for patients with C/C or T/T and C/T genotypes (*p* = 0.023, q = 0.046). Survival curves were plotted using the Kaplan–Meier method and compared using the log-rank test. PFS from treatment start is presented in months.

Modest but significant association with PFS was demonstrated for *ATG10* rs1864183 when analyzed through an overdominant genetic model (C/C + T/T vs. C/T; *p* = 0.023, q = 0.046). The codominant model showed superior PFS in patients with C/C (median PFS 11.01 months) and T/T (median PFS 10.03 months) genotypes compared with C/T carriers (median PFS 6.8 months). However, the difference did not reach statistical significance (*p* = 0.073, q = 0.073) (Figure 2). Other genotypes showed no significant association with PFS and OS in this group of patients.

To identify the most prominent risk factors for progression, univariate Cox proportional hazards screening was performed using the following variables: age at the start of therapy, melanoma localization, histologic subtype, Breslow thickness, mitotic count, the presence of ulcerations, TIL, lymphovascular invasion, tumor regression, *BRAF* status, serum LDH and S-100, inflammatory biomarkers NLR, LMR, MLR, PLR, SII and PIV, number of metastatic sites, ECOG performance status, and *ATG10* rs1864183 genotype in overdominant model. The lymphovascular invasion (*p* < 0.001, q = 0.01), tumor regression (*p* = 0.02, q = 0.028), elevated serum LDH (*p* = 0.005, q = 0.017), and poor ECOG performance status (*p* = 0.003, q = 0.015) were the most prominent risk factors for progression. Table 4 summarizes patient and melanoma characteristics (including the *ATG10* rs1864183 genotype) that significantly impact disease progression in univariate Cox proportional hazards regression.

The impact of elevated serum LDH (HR 3.54, 95% CI 1.14–11; *p* = 0.029), *ATG10* rs1864183 C/T genotype (HR 2.28, 95% CI 1.13–4.63; *p* = 0.022), ECOG > 0 (HR 2.33, 95% CI 1.1–4.94; *p* = 0.027), and PIV index > 277.269 (HR 2.08, 95% CI 1.04–4.17; *p* = 0.038) as independent predictors of progression was confirmed in a multivariate Cox proportional hazards regression analysis that included only significant systemic parameters from the univariate Cox analysis.

## 4. Discussion

In the present study, the clinical impact of *ATG5*, *ATG10*, and *ATG16L1* SNPs in patients with (advanced) melanoma was assessed. Associations of SNPs with baseline histopathologic characteristics were analyzed in the cohort of 144 melanoma patients diagnosed and treated in a single medical center (Military Medical Academy, Belgrade, Serbia). Obtained frequencies of *ATG5* (rs2245214 and rs510432), *ATG10* (rs1864183, rs1864182), and *ATG16L1* (rs2241880) alleles/genotypes were similar to those previously reported for other European populations [18]. In addition, the analyzed *ATG5* rs2245214 and rs510432, as well as *ATG10* rs1864183 and rs1864182 genotypes, were in linkage disequilibrium, as previously reported (NIH, National Cancer Institute, LDlink) [18].

Of note, *ATG5*, *ATG10* and *ATG16L1* encode proteins of ATG12 conjugation system, necessary for autophagosome formation and elongation [19].

The *ATG5* rs2245214 C > G polymorphism is an intronic variant and does not alter the protein’s primary amino acid sequence [18]. Published data reported a lack of association between *ATG5* rs2245214 alleles/genotypes and *ATG5* mRNA expression levels [20]. The *ATG5* rs510432 T > C polymorphism is a variant located in the promoter region, with no annotation in ClinVar [18]. However, previous reports indicated that *ATG5* rs510432 is functionally active and affects mRNA expression. Martin and coworkers demonstrated that variant G (=C in our study) enhanced promoter activity by 23% compared to the A (=T in our study) allele [21]. Similar findings were reported by Ahmad and coworkers, who found significantly higher mRNA expression in asthmatic patients carrying *ATG5* rs510432 C/C than in C/T or T/T carriers [22].

The *ATG10* rs1864183 C > T polymorphism is a missense variant that alters the protein structure (p.Thr212Met) and is in high LD with the *ATG10* rs1864182 C > A polymorphism, which is also a missense variant (p.Pro220His) [18]. In the study of Xie and coworkers on mRNA expression levels in pulmonary tissue, higher expression levels were detected in rs1864182 C/C and rs1864183 G/G (=C/C in our study) carriers [23]. However, Castro and coworkers did not show differences in mRNA levels in cultured peripheral blood mononuclear cells from healthy controls with different *ATG10* rs1864182 genotypes, but they reported a 25% decrease in protein levels in the presence of the G (=C in our study) allele. [24]. *ATG16L1* rs2241880 G > A is a missense variant (p.Thr300Ala) and is a risk factor for inflammatory bowel diseases [18]. Genome-wide association studies (GWAS) showed the association of the *ATG16L1* rs2241880 G allele with Crohn’s Disease [25]. This amino acid substitution makes the protein susceptible to cleavage by caspase 3 [26]. *ATG16L1* rs2241880 was also identified as a risk factor for colorectal carcinoma [27].

The present study assessed the association of *ATG* genotypes with baseline melanoma characteristics and suggested a trend toward an association of *ATG5* rs2245214 with tumor regression and lymphovascular invasion; that is, the C/C genotype was more frequent in patients with unfavorable characteristics (lymphovascular invasion and regression). Also, the *ATG5* rs510432 C/T was more frequent in patients having tumors with ulcerations. The association between *ATG5*, *ATG10* and *ATG16L1* genotypes and pathological characteristics of melanoma at diagnosis was also analyzed in the study of White and coauthors [13]. Their study reported that patients with the *ATG5* rs2245214 C/G or *ATG5* rs510432 C/C genotype were more frequently presented with melanoma in an advanced clinical stage. Also, they found a correlation between the *ATG16L1* rs2241880 G/G genotype and decreased Breslow thickness, and the A/G genotype and younger age at diagnosis.

After the pioneering work of Hu and coworkers on the impact of the systemic Immune-Inflammation index as a predictor of prognosis in cancer patients [28], a significant number of papers provided data about NLR, MLR, PLR, LMR, SII, and PIV as markers for risk stratification in melanoma patients treated with ICIs [29]. Autophagy plays an important role in tumor immune escape and the interplay between the tumor and different leukocyte populations (neutrophils, monocytes, lymphocytes) and platelets, in both the tumor environment and systemic circulation [30]. In our study, *ATG5* rs2245214 was linked to variations in NLR, SII, and PIV. The heterozygous C/G genotype exhibited the lowest median NLR, SII, and PIV scores compared to its homozygous counterparts. *ATG10* rs1864183 genotypes in our study were associated with PIV. The C/C carriers had the lowest, C/T heterozygotes had intermediate, and T/T carriers had the highest PIV. Published data associating immune inflammatory indices with different *ATG5*, *ATG10*, or *ATG16L1* genotypes remain scarce. Due to the limited sample size of our single-center study, further validation in larger patient cohorts is warranted for definitive conclusions.

Elevated LDH levels in parallel with the number of metastases are important biomarkers in advanced melanoma treated with ICI, and they are commonly used to predict tumor burden and prognosis [31,32]. Herein, we report the association of *ATG5* rs2245214 with LDH levels and the number of metastatic sites. Similarly to inflammatory biomarkers, normal LDH levels and up to three metastatic sites were more common in C/G carriers. In the group of patients with available data on best response to therapy, the *ATG5* rs2245214 and *ATG16L1* rs2241880 were associated with therapy response, as a control for disease. A possible explanation may be that ATG5, as a core autophagy protein, is implicated in melanoma progression and, together with ATG16L1, in adaptive resistance to therapy. Previous reports implied an association between lower ATG5 levels and reduced autophagic activity, which contributes to melanoma development and unfavorable outcomes [33]. In our patients with advanced melanoma treated with ICIs, *ATG5* rs2245214 was not associated with progression-free or overall survival.

Among patients with advanced melanoma, those with the *ATG10* rs1864183 C/T genotype had shorter PFS than those with C/C or T/T genotypes. To our knowledge, there are no studies confirming the impact of *ATG10* mRNA expression on clinical progression of cutaneous melanoma treated with ICI as first-line therapy. Also, previous studies reported the impact of *ATG10* polymorphism on therapy resistance and survival in cancer patients. For example, *ATG10* rs1864182 was associated with primary or acquired resistance to Gefitinib in advanced lung adenocarcinoma [34], and both rs1864182 and rs1864183 were associated with poor survival in non-small-cell lung cancer [23].

Study limitations: The present study is based on a relatively small patient cohort, particularly in the subgroup receiving ICIs as first-line treatment. There is substantial missing data on baseline pathologic characteristics of melanoma, e.g., ulceration, lymphovascular invasion, and regression. We included these characteristics in the Cox proportional hazards regression due to their significance in systemic disease.

## 5. Conclusions

Autophagy is an essential process for maintaining cellular homeostasis under stress. It has a significant impact on tumorigenesis, tumor cell survival, and therapy resistance. In advanced melanoma, autophagy interplays among tumor cells, the tumor microenvironment, and different parts of the immune system- cells of innate and adaptive immunity. This highly conserved process has an important impact on tumor cells in terms of adaptation to therapy and suppression of immune cells through different pathways. In our study, we found that *ATG* polymorphisms did not reach statistical significance but showed a trend in association with clinical characteristics of melanoma patients at the time of diagnosis. In advanced melanoma treated with ICIs, *ATG* polymorphisms were associated with systemic inflammation parameters (as presented by NLR, SII, and PIV), tumor burden through LDH levels, and the number of metastatic sites. Finally, resistance to ICIs and PFS were also associated with *ATG* polymorphisms. Therefore, polymorphisms in autophagy may have potential as predictive/prognostic markers in patients with advanced melanoma, in parallel with systemic inflammatory indices, LDH levels, and ECOG performance status. However, we should underline that final conclusions need a larger patient cohort, particularly in the subgroup with unresectable melanoma receiving first-line immunotherapy. In addition, further research should separate the ICI group into those receiving PD-1/PD-L1 inhibitors, CTLA-4 inhibitors and dual ICI therapy.

## Figures and Tables

**Table 1:** Baseline characteristics of patients/melanomas.

Characteristics	Whole Group *n* (%)	First-Line ICIs *n* (%)
**Gender**		
Male	91 (63.19)	47 (63.51)
Female	53 (36.81)	27 (36.49)
**Age at diagnosis**		
≤60 years	70 (48.61)	31 (41.89)
>60 years	74 (51.39)	43 (58.11)
**Histologic subtype**		
Superficial spreading melanoma	71 (49.31)	35 (47.3)
Nodular melanoma	39 (27.08)	22 (29.73)
Lentigo maligna melanoma	1 (0.69)	1 (1.35)
Acral lentiginous melanoma	5 (3.47)	3 (4.05)
Other	28 (19.44)	13 (17.57)
**Melanoma localization**		
Head&neck	23 (15.97)	15 (20.27)
Trunk	57 (39.58)	28 (37.84)
Upper extremities	15 (10.42)	10 (13.51)
Lower extremities	35 (24.31)	13 (17.57)
Other/unknown	14 (9.72)	8 (10.81)
**Clinical stage ^1^**		
I	11 (7.64)	0
II	36 (25)	0
III	72 (50)	10 (13.51)
IV	21 (14.58)	64 (86.49)
Unknown/missing	4 (2.78)	0
**Breslow thickness**		
<4 mm	68 (47.22)	29 (39.19)
≥4 mm	51 (35.42)	30 (40.54)
Unknown/missing	25 (17.36)	15 (20.27)
**Ulcerations**		
Present	48 (33.33)	45 (60.81)
Absent	68 (47.22)	15 (20.27)
Unknown/missing	28 (19.44)	14 (18.92)
**Mitotic rate**		
≤5	32 (22.22)	17 (22.97)
>5	56 (38.89)	25 (33.78)
Unknown/missing	56 (38.89)	32 (43.24)
**TIL ^2^**		
Present	65 (45.14)	33 (44.59)
Absent	3 (2.08)	3 (4.05)
Unknown/missing	76 (52.78)	38 (51.35)
**Microsatellites**		
Present	6 (4.17)	0
Absent	138 (95.83)	74 (100)
**Tumor regression**		
Present	13 (9.03)	7 (9.46)
Absent	40 (27.78)	15 (20.27)
Unknown/missing	91 (63.19)	52 (70.27)
**Lymphovascular invasion**		
Present	11 (7.64)	4 (5.41)
Absent	62 (43.05)	32 (43.24)
Unknown/missing	71 (49.31)	38 (51.35)
**Perineural invasion**		
Present	1 (0.69)	1 (1.35)
Absent	57 (39.58)	26 (35.14)
Unknown/missing	86 (59.72)	47 (63.51)
***BRAF*** **mutation status**		
Positive	77 (53.47)	41 (55.41)
Negative	63 (43.75)	32 (43.24)
Unknown/missing	4 (2.78)	1 (1.35)

^1^ Clinical stage at baseline in the total patient cohort (*n* = 144), and at the start of therapy in patients with advanced melanoma treated with first-line immunotherapy (*n* = 74); ^2^ TIL, Tumor Infiltrating Lymphocytes.

**Table 2:** Response to therapy in patients with unresectable melanoma in clinical stage III/IV treated with first-line ICIs.

Clinical Outcome	Description/RECIST 1.1 Criteria	*n* (%)
Disease Control Rate (DCR)	Primary Progressive Disease (PD)	15 (22.39)
	Disease Control (CR + PR + MR + SD)	52 (77.61)
Objective Response Rate (ORR)	Non-Responders (SD + MR + PD)	34 (50.75)
	Objective Responders (CR + PR)	33 (49.25)
Missing Data	Non-evaluable/Missing Response Data	7 (9.46%)

RECIST 1.1, Response Evaluation Criteria in Solid Tumors version 1.1; PD, progressive disease; CR, complete response; PR, partial response; MR, mixed response; SD, stable disease.

**Table 3:** Association of *ATG5* rs2245214 and *ATG16L1* rs2241880 genotypes with disease control rate in patients with unresectable melanoma in clinical stage III/IV treated with first-line ICIs.

Gene/Polymorphism	Genetic Model	Genotype	PD ^1^ *n* (%)	CR + PR + MR + SD ^2^ *n* (%)	*p* ^3^	FDR q ^4^ Values
*ATG5* rs2245214	codominant	C/C C/G G/G	11 (73.3%) 3 (20.0%) 1 (6.7%)	17 (32.7%) 30 (57.7%) 5 (9.6%)	0.013	0.029
*ATG5* rs2245214	dominant	C/C C/G + G/G	11 (73.3%) 4 (26.7%)	17 (32.7%) 35 (67.3%)	0.007	0.029
*ATG5* rs2245214	overdominant	C/C + G/G C/G	12 (80.0%) 3 (20.0%)	22 (42.3%) 30 (57.7%)	0.017	0.029
*ATG16L1* rs2241880	codominant	A/A A/G G/G	7 (46.7%) 7 (46.7%) 1 (6.7%)	12 (23.1%) 20 (38.5%) 20 (38.5%)	0.032	0.032
*ATG16L1* rs2241880	dominant	A/G + A/A G/G	14 (93.3%) 1 (6.7%)	32 (61.5%) 20 (38.5%)	0.026	0.032

^1^ PD, progressive disease. ^2^ CR, complete response; PR, partial response; MR, mixed response; SD, stable disease. ^3^ *p*-values were obtained by Fisher’s exact probability test. ^4^ q values were calculated using Benjamini–Hochberg False Discovery Rate (FDR).

**Table 4:** The most prominent risk factors for progression in patients with unresectable melanoma in clinical stage III/IV treated with first-line immunotherapy (univariate Cox proportional hazards screening).

Characteristic/Risk Factor	HR (95% CI)	*p*-Value	q-Value
Lymphovascular invasion (present)	12.72 (3.05–52.99)	<0.001	0.01
Tumor regression (present)	3.77 (1.24–11.49)	0.02	0.028
LDH (elevated)	2.91 (1.38–6.12)	0.005	0.017
ECOG (>0)	2.76 (1.41–5.4)	0.003	0.015
Number of metastatic sites (>3)	2.26 (1.12–4.54)	0.023	0.028
NLR (>3.748)	2.23 (1.06–4.68)	0.034	0.034
SII (>763.958)	2.19 (1.16–4.14)	0.016	0.028
PIV (>277.269)	2.14 (1.17–3.92)	0.014	0.028
*ATG10* rs1864183 C/T	1.87 (1.08–3.22)	0.025	0.028
LMR (>3.351)	0.46 (0.25–0.84)	0.011	0.028

HR, hazard ratio.

## Data Availability

The data underlying this article will be shared on reasonable request to the PI of the ReDiMEL project (lkandolfsekulovic@gmail.com).

## Supplementary Materials

The following supporting information can be downloaded at: https://www.mdpi.com/article/10.3390/cancers18162709/s1, Table S1: The frequencies of alleles and genotypes in patients with melanoma (*n* = 144).

## Abbreviations

The following abbreviations are used in this manuscript:

ICIs	Immune Checkpoint Inhibitors
PD-1	Programmed Death-1
PD-L1	Programmed Death-Ligand-1
CTLA-4	Cytotoxic T-Lymphocyte Antigen-4
LDH	Lactate Dehydrogenase
ECOG	Eastern Cooperative Oncology Group
ATG	Autophagy-related gene
SNPs	Single Nucleotide Polymorphisms
PFS	Progression-Free Survival
OS	Overall Survival
OR	Odds Ratio
CI	Confidence Interval
LMR	Lymphocyte-to-Monocyte Ratio
NLR	Neutrophil-to-Lymphocyte Ratio
PLR	Platelet-to-Lymphocyte Ratio
MLR	Monocyte-to-Lymphocyte Ratio
PIV	Pan-Immune-inflammation Value
SII	Systemic Immune-Inflammation
TIL	Tumor Infiltrating Lymphocyte
HR	Hazard Ratio
GWAS	Genome-Wide Association Study

## References

[1] Garbe C., Amaral T., Peris K., Hauschild A., Arenberger P., Basset-Seguin N., Bastholt L., Bataille V., Brochez L., del Marmol V. (2025). European Consensus-Based Interdisciplinary Guideline for Melanoma. Part 2: Treatment–Update 2024. Eur. J. Cancer.

[2] Boutros A., Croce E., Ferrari M., Gili R., Massaro G., Marconcini R., Arecco L., Tanda E.T., Spagnolo F. (2024). The Treatment of Advanced Melanoma: Current Approaches and New Challenges. Crit. Rev. Oncol./Hematol..

[3] Wang J., Chen Q., Shan Q., Liang T., Forde P., Zheng L. (2025). Clinical Development of Immuno-Oncology Therapeutics. Cancer Lett..

[4] Amaral T., Ottaviano M., Arance A., Blank C., Chiarion-Sileni V., Donia M., Dummer R., Garbe C., Gershenwald J.E., Gogas H. (2025). Cutaneous Melanoma: ESMO Clinical Practice Guideline for Diagnosis, Treatment and Follow-Up. Ann. Oncol..

[5] Mehnert J.M., Mitchell T.C., Huang A.C., Aleman T.S., Kim B.J., Schuchter L.M., Linette G.P., Karakousis G.C., Mitnick S., Giles L. (2022). BAMM (BRAF Autophagy and MEK Inhibition in Melanoma): A Phase I/II Trial of Dabrafenib, Trametinib, and Hydroxychloroquine in Advanced BRAFV600-Mutant Melanoma. Clin. Cancer Res. Off. J. Am. Assoc. Cancer Res..

[6] Singh S.S., Vats S., Chia A.Y.Q., Tan T.Z., Deng S., Ong M.S., Arfuso F., Yap C.T., Goh B.C., Sethi G. (2018). Dual Role of Autophagy in Hallmarks of Cancer. Oncogene.

[7] Foth M., McMahon M. (2021). Autophagy Inhibition in BRAF-Driven Cancers. Cancers.

[8] Pangilinan C., Klionsky D.J., Liang C. (2024). Emerging Dimensions of Autophagy in Melanoma. Autophagy.

[9] Shukla S.A., Bachireddy P., Schilling B., Galonska C., Zhan Q., Bango C., Langer R., Lee P.C., Gusenleitner D., Keskin D.B. (2018). Cancer-Germline Antigen Expression Discriminates Clinical Outcome to CTLA-4 Blockade. Cell.

[10] Jalouli M., Harrath A.H., Al-Zharani M., Rahman M.A. (2026). Autophagy Modulation in Cancer Immunotherapy, Emerging Molecular Targets and Drug Selection Strategies. Int. J. Mol. Sci..

[11] Tang H., You T., Ge H., Bai C., Wang Y., Sun Z., Han Q., Zhao R.C. (2025). Autophagy Inhibition Improves the Efficacy of Anlotinib and PD-1 Inhibitors in the Treatment of NSCLC. J. Immunother. Cancer.

[12] Grosjean I., Roméo B., Domdom M.A., Belaid A., D’Andréa G., Guillot N., Gherardi R.K., Gal J., Milano G., Marquette C.H. (2022). Autophagopathies: From Autophagy Gene Polymorphisms to Precision Medicine for Human Diseases. Autophagy.

[13] White K.A.M., Luo L., Thompson T.A., Torres S., Hu C.-A.A., Thomas N.E., Lilyquist J., Anton-Culver H., Gruber S.B., From L. (2016). Variants in Autophagy-Related Genes and Clinical Characteristics in Melanoma: A Population-Based Study. Cancer Med..

[14] Eisenhauer E.A., Therasse P., Bogaerts J., Schwartz L.H., Sargent D., Ford R., Dancey J., Arbuck S., Gwyther S., Mooney M. (2009). New Response Evaluation Criteria in Solid Tumours: Revised RECIST Guideline (Version 1.1). Eur. J. Cancer.

[15] Dujovic B., Popovic A., Jalovcic Suljevic A., Cikota-Aleksic B., Balic M., Salatic I., Pavlica J., Schnecko P., Mesti T., Terzo M. (2026). Prognostic Significance of Inflammatory Biomarkers in First-Line Immunotherapy for Metastatic Melanoma: Multicentric Study. Cancers.

[16] Solé X. SNPStats: Your Web Tool for SNP Analysis. https://www.snpstats.net/start.htm.

[17] Kanda Y. EZR (Easy R): Free Statistical Software. https://www.jichi.ac.jp/usr/hema/EZR/windowsEN.html.

[18] National Center for Biotechnology Information (NCBI). https://www.ncbi.nlm.nih.gov/.

[19] Mizushima N., Yoshimori T., Ohsumi Y. (2011). The Role of Atg Proteins in Autophagosome Formation. Annu. Rev. Cell Dev. Biol..

[20] Ciccacci C., Perricone C., Alessandri C., Latini A., Politi C., Delunardo F., Pierdominici M., Conti F., Novelli G., Ortona E. (2018). Evaluation of ATG5 Polymorphisms in Italian Patients with Systemic Lupus Erythematosus: Contribution to Disease Susceptibility and Clinical Phenotypes. Lupus.

[21] Martin L.J., Gupta J., Jyothula S.S.S.K., Kovacic M., Myers J.M., Patterson T.L., Ericksen M.B., He H., Gibson A.M., Baye T.M. (2012). Functional Variant in the Autophagy-Related 5 Gene Promotor Is Associated with Childhood Asthma. PLoS ONE.

[22] Ahmad E.S., Diab S.M., Behiry E.G., El Bassyoni S.E.B.E.S., Ishak S.R., Ramadan A. (2022). Autophagy-Related 5 Gene MRNA Expression and ATG5 Rs510432 Polymorphism in Children with Bronchial Asthma. Pediatr. Pulmonol..

[23] Xie K., Liang C., Li Q., Yan C., Wang C., Gu Y., Zhu M., Du F., Wang H., Dai J. (2016). Role of ATG10 Expression Quantitative Trait Loci in Non-Small Cell Lung Cancer Survival. Int. J. Cancer.

[24] Castro I., Sampaio-Marques B., Areias A.C., Sousa H., Fernandes Â., Sanchez-Maldonado J.M., Cunha C., Carvalho A., Sainz J., Ludovico P. (2021). Functional Genetic Variants in ATG10 Are Associated with Acute Myeloid Leukemia. Cancers.

[25] Hampe J., Franke A., Rosenstiel P., Till A., Teuber M., Huse K., Albrecht M., Mayr G., De La Vega F.M., Briggs J. (2007). A Genome-Wide Association Scan of Nonsynonymous SNPs Identifies a Susceptibility Variant for Crohn Disease in ATG16L1. Nat. Genet..

[26] Murthy A., Li Y., Peng I., Reichelt M., Katakam A.K., Noubade R., Roose-Girma M., Devoss J., Diehl L., Graham R.R. (2014). A Crohn’s Disease Variant in Atg16l1 Enhances Its Degradation by Caspase 3. Nature.

[27] Cao H., Li Z., Zhou D., Wan L., Yu D., Zhang J., Xu E., Zhang D., Lai M. (2016). ATG16L1 Rs2241880 Polymorphism Predicts Unfavorable Clinical Outcomes for Colorectal Cancer Patients in the Chinese Population. Int. J. Clin. Exp. Pathol..

[28] Hu B., Yang X.R., Xu Y., Sun Y.F., Sun C., Guo W., Zhang X., Wang W.M., Qiu S.J., Zhou J. (2014). Systemic Immune-Inflammation Index Predicts Prognosis of Patients after Curative Resection for Hepatocellular Carcinoma. Clin. Cancer Res..

[29] Susok L., Said S., Reinert D., Mansour R., Scheel C.H., Becker J.C., Gambichler T. (2022). The Pan-Immune-Inflammation Value and Systemic Immune-Inflammation Index in Advanced Melanoma Patients under Immunotherapy. J. Cancer Res. Clin. Oncol..

[30] Wang H., Sun P., Yuan X., Xu Z., Jiang X., Xiao M., Yao X., Shi Y. (2025). Autophagy in Tumor Immune Escape and Immunotherapy. Mol. Cancer.

[31] Janka E.A., Ványai B., Szabó I.L., Toka-Farkas T., Várvölgyi T., Kapitány A., Szegedi A., Emri G. (2023). Primary Tumour Category, Site of Metastasis, and Baseline Serum S100B and LDH Are Independent Prognostic Factors for Survival in Metastatic Melanoma Patients Treated with Anti-PD-1. Front. Oncol..

[32] Hill C.N., Hernández-Cáceres M.P., Asencio C., Torres B., Solis B., Owen G.I. (2020). Deciphering the Role of the Coagulation Cascade and Autophagy in Cancer-Related Thrombosis and Metastasis. Front. Oncol..

[33] Frangež Ž., Gérard D., He Z., Gavriil M., Fernández-Marrero Y., Seyed Jafari S.M., Hunger R.E., Lucarelli P., Yousefi S., Sauter T. (2021). ATG5 and ATG7 Expression Levels Are Reduced in Cutaneous Melanoma and Regulated by NRF1. Front. Oncol..

[34] Yuan J., Zhang N., Yin L., Zhu H., Zhang L., Zhou L., Yang M. (2017). Clinical Implications of the Autophagy Core Gene Variations in Advanced Lung Adenocarcinoma Treated with Gefitinib. Sci. Rep..

