# Supplementary material for: Variations in Autophagy-Related Genes ATG5, ATG10 and ATG16L1 Correlate with Tumor Burden, Inflammatory Biomarkers and Clinical Course in Patients with Metastatic Melanoma Treated with Immune Checkpoint Inhibitors
Source: Cancers (Basel). 2026 Aug 21;18(16):2709. doi: 10.3390/cancers18162709 (PMC13511534; doi:10.3390/cancers18162709)
Supplement: Supplementary file 1 [file cancers-18-02709-s001.zip › cancers-4444689-supplementary.pdf]

Supplementary Table S1. The frequencies of alleles and genotypes in patients with melanoma (*n* = 144).

***ATG5* rs2245214**			
Allele frequencies		Genotype frequencies	
		C/C	0.46
**C**	0.68	C/G	0.44
G	0.32	G/G	0.10
Exact test for Hardy-Weinberg equilibrium		p=0.85	
***ATG5* rs510432**			
Allele frequencies		Genotype frequencies	
		T/T	0.31
**T**	0.56	T/C	0.50
C	0.44	C/C	0.19
Exact test for Hardy-Weinberg equilibrium		p=1.0	
***ATG10* rs1864183**			
Allele frequencies		Genotype frequencies	
		C/C	0.26
**C**	0.50	C/T	0.48
T	0.50	T/T	0.26
Exact test for Hardy-Weinberg equilibrium		p=0.51	
***ATG10* rs1864182**			
Allele frequencies		Genotype frequencies	
		C/C	0.21
**C**	0.43	A/C	0.45
A	0.57	A/A	0.34
Exact test for Hardy-Weinberg equilibrium		p=0.50	
***ATG16L1* rs2241880**			
Allele frequencies		Genotype frequencies	
		G/G	0.29
**G**	0.52	A/G	0.46
A	0.48	A/A	0.25
Exact test for Hardy-Weinberg equilibrium		p=0.32	
